# Modeling clonal structure over narrow time frames via circulating tumor DNA in metastatic breast cancer

**DOI:** 10.1186/s13073-021-00895-x

**Published:** 2021-05-20

**Authors:** Zachary T. Weber, Katharine A. Collier, David Tallman, Juliet Forman, Sachet Shukla, Sarah Asad, Justin Rhoades, Samuel Freeman, Heather A. Parsons, Nicole O. Williams, Romualdo Barroso-Sousa, Elizabeth H. Stover, Haider Mahdi, Carrie Cibulskis, Niall J. Lennon, Gavin Ha, Viktor A. Adalsteinsson, Sara M. Tolaney, Daniel G. Stover

**Affiliations:** 1 The Ohio State University Comprehensive Cancer Center, The Ohio State University, 460 W. 12th Avenue, Columbus, OH 43210 USA; 2 Division of Medical Oncology, Department of Medicine, College of Medicine, The Ohio State University, 320 W. 10th Avenue, Columbus, OH 43210 USA; 3 Broad Institute of Harvard & MIT, 415 Main St., Cambridge, MA 02412 USA; 4 Department of Medical Oncology, Dana-Farber Cancer Institute, 450 Brookline Avenue, Boston, MA 02215 USA; 5 Translational Immunogenomics Lab, Dana-Farber Cancer Institute, 450 Brookline Avenue, Boston, MA 02215 USA; 6 Hospital Sirio-Libanes, SGAS 613/614, Conjunto E, Lote 95, Brasília, Brazil; 7 Department of Obstetrics and Gynecology, Cleveland Clinic, Cleveland, OH 44195 USA; 8 Department of Surgery, Case Comprehensive Cancer Center, Cleveland, OH 44106 USA; 9 Public Health Sciences Division, Fred Hutchinson Cancer Research Center, Seattle, WA 98109 USA; 10 Biomedical Research Tower, Room 984, Ohio State University Comprehensive Cancer Center, Stefanie Spielman Comprehensive Breast Center, Columbus, OH 43210 USA

**Keywords:** ctDNA, Circulating tumor DNA, Tumor evolution, Neoantigens, Serial sequencing, Ultra-low pass whole genome sequencing, Targeted panel sequencing, Liquid biopsy

## Abstract

**Background:**

Circulating tumor DNA (ctDNA) offers minimally invasive means to repeatedly interrogate tumor genomes, providing opportunities to monitor clonal dynamics induced by metastasis and therapeutic selective pressures. In metastatic cancers, ctDNA profiling allows for simultaneous analysis of both local and distant sites of recurrence. Despite the promise of ctDNA sampling, its utility in real-time genetic monitoring remains largely unexplored.

**Methods:**

In this exploratory analysis, we characterize high-frequency ctDNA sample series collected over narrow time frames from seven patients with metastatic triple-negative breast cancer, each undergoing treatment with Cabozantinib, a multi-tyrosine kinase inhibitor (NCT01738438, https://clinicaltrials.gov/ct2/show/NCT01738438). Applying orthogonal whole exome sequencing, ultra-low pass whole genome sequencing, and 396-gene targeted panel sequencing, we analyzed 42 plasma-derived ctDNA libraries, representing 4–8 samples per patient with 6–42 days between samples. Integrating tumor fraction, copy number, and somatic variant information, we model tumor clonal dynamics, predict neoantigens, and evaluate consistency of genomic information from orthogonal assays.

**Results:**

We measured considerable variation in ctDNA tumor faction in each patient, often conflicting with RECIST imaging response metrics. In orthogonal sequencing, we found high concordance between targeted panel and whole exome sequencing in both variant detection and variant allele frequency estimation (specificity = 95.5%, VAF correlation, *r* = 0.949), Copy number remained generally stable, despite resolution limitations posed by low tumor fraction. Through modeling, we inferred and tracked distinct clonal populations specific to each patient and built phylogenetic trees revealing alterations in hallmark breast cancer drivers, including *TP53, PIK3CA, CDK4*, and *PTEN*. Our modeling revealed varied responses to therapy, with some individuals displaying stable clonal profiles, while others showed signs of substantial expansion or reduction in prevalence, with characteristic alterations of varied literature annotation in relation to the study drug. Finally, we predicted and tracked neoantigen-producing alterations across time, exposing translationally relevant detection patterns.

**Conclusions:**

Despite technical challenges arising from low tumor content, metastatic ctDNA monitoring can aid our understanding of response and progression, while minimizing patient risk and discomfort. In this study, we demonstrate the potential for high-frequency monitoring of evolving genomic features, providing an important step toward scalable, translational genomics for clinical decision making.

**Supplementary Information:**

The online version contains supplementary material available at 10.1186/s13073-021-00895-x.

## Background

Tumors are known to shed fragments of DNA into the bloodstream through apoptosis and necrosis [1–3]. This cell-free DNA, known as circulating tumor DNA (ctDNA), can be acquired minimally invasively through simple blood draws, then isolated from plasma in admixture with cell-free DNA of non-tumor origin. The potential for minimally invasive tumor profiling makes ctDNA an attractive target for biomarker development and serial profiling, especially in metastatic cancers. Despite relative ease of collection, ctDNA assays are challenging due to lower purity relative to tumor tissue samples. For example, estimated ctDNA purity, or tumor fraction (TFx), ranges from <0.01 to 0.80 in large cohorts of metastatic cancer, with most samples with a TFx <0.10 and varying by cancer type [4].

Despite technical challenges of ctDNA, progress has been made in recent years in leveraging plasma samples for clinical and genomic applications using diverse sequencing approaches, including specific mutation tracking, targeted panel sequencing, shallow whole genome sequencing, methylation, and whole exome/genome sequencing. PCR-based strategies demonstrated the ability to precisely track and quantify known variants in metastatic breast cancer [5, 6]. Exome-based and targeted panel sequencing strategies have suggested high concordance between alterations discovered in circulating tumor DNA [4], circulating tumor cells [7], and matched tumor biopsies in solid tumors [8] and blood cancers, like multiple myeloma [9, 10], where cancer cells are difficult to reach without bone marrow biopsy. Importantly, ctDNA profiles have also demonstrated the capability to capture novel somatic alterations not present in primary cancers [4, 11, 12]. In metastatic cancer, ctDNA may act as a “sink” of tumor DNA from multiple metastatic sites from which genetic alterations across multiple sites may be simultaneously profiled [13–15]. Further, ctDNA tumor fraction levels have been found to correlate with patient outcomes [9, 11, 16–18], pointing to a potential for broader clinical application of ctDNA assays. Many potential applications are under development, including cancer screening [19], minimal residual disease assessment [20–23], and tumor monitoring [18].

Circulating tumor DNA analyses offer the potential to monitor tumor genomic features over more narrow time windows, on the order of days to weeks or less, than is logistically or ethically feasible with repeated tissue biopsies. An outstanding question in oncology, and specifically the ctDNA field, is how rapidly tumor genomes evolve under therapeutic selective pressures, and whether this can be detected via ctDNA through the growing number of sequencing approaches. To evaluate this question, we focused on triple-negative breast cancer (TNBC), an aggressive form of breast cancer defined by the lack of expression of three clinically important therapeutic targets, the ER, PR, and HER2 receptors [24]. Metastatic TNBC (mTNBC) is known to shed relatively high amounts of ctDNA [11]. TNBC constitutes around 10–15% of all breast cancer, but may be responsible for upwards of 30% of breast cancer mortality [24–26].

In this work, we provide the first comprehensive analysis of ctDNA genetic profiling over narrow time windows in mTNBC. We leverage serial sets of ctDNA collected from patients with mTNBC enrolled in a phase II clinical trial of Cabozantinib, a multi-receptor tyrosine kinase inhibitor, as an exploratory analysis of available samples. These clinical trial samples, whose primary endpoints were previously reported [27], provide a cohort of patients on a uniform and targeted treatment regimen. Using orthogonal sequencing approaches, we demonstrate the feasibility of ctDNA genetic profiling for modeling pan-tumor clonal dynamics, rare variant detection, copy number analysis, and neoantigen prediction. This work was presented in part as a conference abstract [28].

## Methods

### Patient eligibility, selection, and treatment

Individuals were considered eligible for study if they were 18 years of age or older with diagnosed TNBC, designated by the following indications: estrogen receptor-negative (ER−; <10% staining by immunohistochemistry [IHC]), progesterone receptor-negative (PR−; <10% staining by IHC), and HER2-negative (0 or 1+ by IHC or fluorescence in situ hybridization [FISH] ratio<2.0). Patients had measurable disease by Response Evaluation Criteria In Solid Tumors (RECIST) version 1.1 and may have received 0 to 3 prior chemotherapeutic regimens for mTNBC. Key exclusion criteria include the following: receiving another investigational agent within 2 weeks of the first dose of cabozantinib, untreated brain metastases, symptomatic brain metastases, or those which required therapy for symptom control, or prior treatment with a MET inhibitor (other than tivantinib ARQ-197) [27].

Patients who met eligibility criteria and consented to participation were enrolled in a single-arm, two-stage phase II study assessing the efficacy of cabozantinib monotherapy in patients with mTNBC (NCT01738438, https://clinicaltrials.gov/ct2/show/NCT01738438). Treatment consisted of oral dosing of cabozantinib at 60mg daily over a 21-day cycle. Patients underwent radiographic restaging at 6 weeks and every 9 weeks thereafter. Patients were enrolled from February 2013 to May 2015. The primary endpoint was the activity of cabozantinib, as defined by objective response rate (ORR) in patients with mTNBC. Predefined secondary endpoints included progression-free survival (PFS), toxicity, and pain. Correlative studies included analysis of MET and phospho-MET expression in archival tumor tissue, and molecular and cellular biomarkers of cabozantinib. The results of this study have been published previousl y[27]. The analyses presented herein are exploratory analyses of existing plasma specimens. Clinicopathologic data were abstracted from the medical record. Research was approved by local human research protections programs and institutional review boards at the Dana-Farber Cancer Institute and Ohio State University, and studies were conducted in accordance with the Declaration of Helsinki.

### Sample collection and processing

Plasma was collected at baseline, on day 8 of therapy, on day 1 of each 21-day cycle of therapy, and, if available, at the time of progression. Eight milliliters of the blood was collected in BD brand EDTA vacutainers and processed within 4 h of collection at the Clinical Laboratory Improvement Amendments-certified core in the Steele Laboratories (Massachusetts General Hospital), where the whole blood was separated into cellular fraction and plasma by centrifuging at 1000–1900×*g* for 10 min at room temperature. Plasma was stored at −80°C.

### Extraction and quantification of cfDNA and germline DNA

Frozen aliquots of the plasma were thawed at room temperature then centrifuged a second time at 15,000×*g* for 10 min at room temperature in low-bind tubes to remove residual cells from plasma. cfDNA was extracted from 1 to 7 mL of plasma and eluted into 40–80 μL of re-suspension buffer using the Qiagen Circulating DNA kit on the QIAsymphony liquid handling system. Germline DNA (gDNA) was extracted from 400 μL of the blood and eluted into 200 μL of re-suspension buffer using the Qiasymphony DSP DNA midi kit on the QIAsymphony liquid handling system. Extracted cfDNA and gDNA was frozen at −20 °C until ready for further processing. Quantification of extracted cfDNA and gDNA was performed using the PicoGreen (Life Technologies) assay on a Hamilton STAR-line liquid handling system.

### Library construction of cfDNA and gDNA

For cfDNA, initial DNA input was normalized to the range 25–52.5 ng in 50 uL of TE buffer (10mM Tris HCl 1mM EDTA, pH 8.0) according to picogreen quantification. For gDNA, an aliquot of gDNA (50–200ng in 50μL) was used as the input into DNA fragmentation (aka shearing). Shearing was performed acoustically using a Covaris focused-ultrasonicator, targeting 150bp fragments. Library preparation was performed using a commercially available kit provided by KAPA Biosystems (KAPA HyperPrep Kit with Library Amplification product KK8504) and IDT’s duplex UMI adapters. Unique 8-base dual index sequences embedded within the p5 and p7 primers (purchased from IDT) were added during PCR. Enzymatic clean-ups were performed using Beckman Coultier AMPure XP beads with elution volumes reduced to 30μL to maximize library concentration. Library quantification was performed using the Invitrogen Quant-It broad range dsDNA quantification assay kit (Thermo Scientific Catalog: Q33130).

### In-solution hybrid selection for exome or targeted panels

After library construction, hybridization and capture were performed using the relevant components of IDT’s XGen hybridization and wash kit and following the manufacturer’s suggested protocol, with several exceptions. A set of 12-plex pre-hybridization pools were created. Custom exome bait (TWIST Biosciences) along with hybridization mastermix was added to the lyophilized pre-hybridization pool prior to resuspension. Library normalization and hybridization setup were performed on a Hamilton Starlet liquid handling platform, while target capture was performed on the Agilent Bravo automated platform. Post capture, a PCR was performed to amplify the capture material. After post-capture enrichment, library pools were quantified using qPCR (automated assay on Agilent Bravo), using a kit purchased from KAPA Biosystems with probes specific to the ends of the adapters. Based on qPCR quantification, pools were normalized using a Hamilton Starlet to 2nM and sequenced using Illumina sequencing technology. The targeted panel bait set used in this study was designed at the Broad Institute to maximize pan-cancer utility and contains regions from 396 driver genes previously annotated in cancer literature.

### Cluster amplification and sequencing

Cluster amplification of library pools was performed according to the manufacturer’s protocol (Illumina) using the Exclusion Amplification cluster chemistry and HiSeq X flowcells. Flowcells were sequenced on v2 Sequencing-by-Synthesis chemistry for HiSeq X flowcells. The flowcells were then analyzed using RTA v.2.7.3 or later. Each pool of libraries was run on paired 151bp runs, reading the dual-indexed sequences to identify molecular indices and sequenced across the number of lanes needed to meet coverage for all libraries in the pool. For ultra-low-pass whole genome sequencing (ULP-WGS), we sequenced cfDNA to an average genome-wide fold coverage of ∼0.1X.

### Tumor fraction, purity, and ploidy assessment of cfDNA

For ULP-WGS, we applied ichorCNA [4], a software package which simultaneously predicts regions of CNAs and estimates the fraction of tumor in ULP-WGS. The workflow consists of three steps: first, computation of read coverage over binned 1 MB genomic regions, next, normalization of coverage to known sources of bias, and finally joint inference of the CNA profile and estimation of tumor fraction.

### Variant calling and copy number assessment

Somatic SNV and INDEL calling in both WES and TPS were completed on the Terra/Firecloud platform using gatk-Mutect2 pipelines (https://portal.firecloud.org/?return=terra#methods/getzlab/CGA_WES_Characterization_Pipeline_v0.1_Dec2018/2) [29, 30]. With exome sequencing, we employed the standard Mutect2 tools, including the orientation-bias filtering model provided in GATK-4.1.6.0. Taking advantage of the serial design of our study, we leveraged Mutect2 Multi-sample mode to borrow information across samples belonging to the same patient, for local haplotype reassembly. Panel sequencing variants were delivered by the Broad Institute who employed tools in GATK-4.1.0.0 with liquid biopsy and duplex-UMI sequencing-specific parameters.

To compare purity and ploidy information from WES to that of ULP-WGS/ichorCNA, we implemented ABSOLUTE [31] and FACETS [32]. ABSOLUTE was run as described via the CGA WES characterization pipeline, developed by the Getz Lab (see above). For FACETS, which requires a database of common SNP locations, we chose the dbSNP release 138 [32] for hg19 aligned sequencing. Finally, for correlation studies of log-ratio, we employed CNVkit [33], a copy number profiling tool which relies on target level read count binning and circular binary segmentation.

### Clonal dynamics and phylogenetic reconstruction

To model the clonal structure and dynamics of metastatic breast cancer, we employed the popular python-based tool, PyClone [34], to use hierarchical-Bayes techniques for jointly estimating prevalence of somatic alterations and simultaneously clustering them into groups representing the underlying cancer’s cell population structure. PyClone inputs require read count information for somatic alterations, as well as their copy number state and sample purity. For our variant sets, we chose the union of filter-passing alterations from each sampled time point delivered by the commercially available liquid-biopsy targeted panel-sequencing pipeline at the Broad Institute. In addition to this set, we added the filter passing alterations discovered through orthogonal exome sequencing, so long as they intersected the 396-gene panel bait-target regions. For copy number information, we intersected our genomic variants with the discrete states determined in ichorCNA profiles at baseline and used the corresponding total_copy_number settings for the preparation of genotype files. ichorCNA also provided sample-level estimates of purity. We chose the PyClone Binomial model, with standard concentration and base measure parameters for the MCMC process. Each patient model was run for 15,000 iterations with the initial 1500 steps thrown out as burn-in. Sequencing error rate for our TPS-based data was set to 0.001, based on earlier estimates from the panel developers. Phylogenetic tree inferences were made using PyClone estimates of the prevalence and the CITUP-QIP algorithm [35], choosing the optimal tree for further investigation of biological context.

### Neoantigen prediction

Neoantigen-binding predictions for known MHC molecules were completed using machine learning approaches learned on peptide-affinity data, NetMHCpan 4.0 [36]. We set scoring thresholds at 0.5% for strong binders and 2.0% for weak binders, representing the rank of the prediction against a panel of random natural peptide sequences, as described by NetMHCpan.

### Statistical tests and data visualization

Figure plotting and statistical tests were completed in R 3.6.3, with heatmaps generated by the ComplexHeatmap Package [37]. All *T* tests were performed with unequal variance procedures using the Welch–Satterthwaite approximation for degrees of freedom.

## Results

### Metastatic TNBC patient and sample selection

**Fig. 1:**
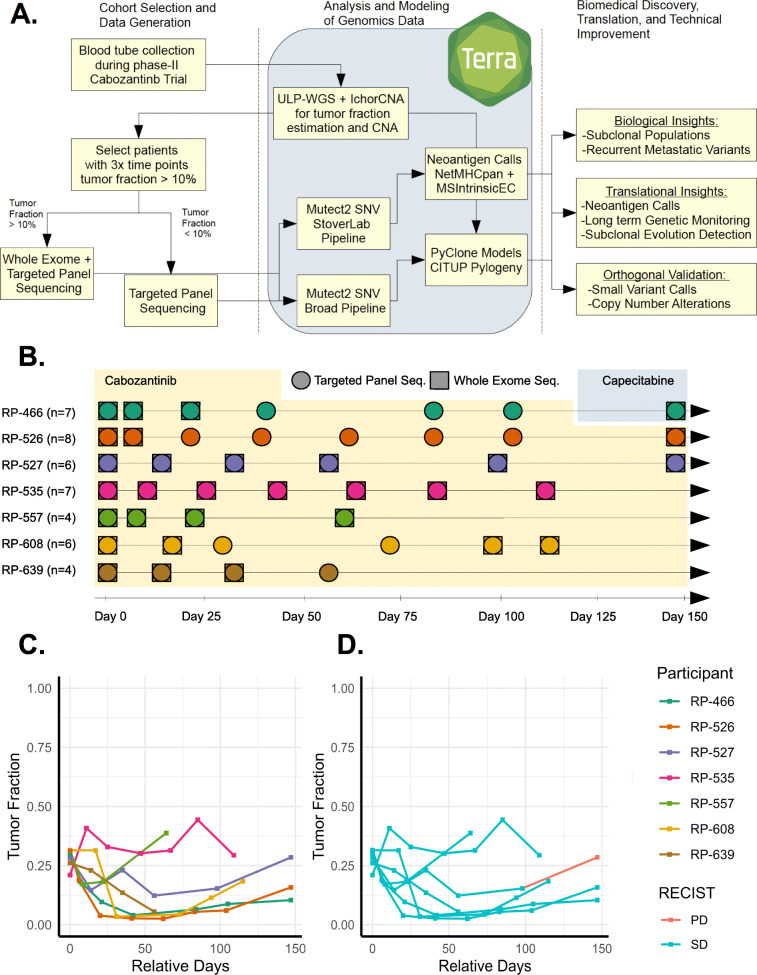
Study design and sampling dynamics. **a** Schematic diagram of the analysis workflow from patient selection, sample capture, and sequencing to downstream analyses. We leveraged the Terra Genomics/FireCloud platform for data storage and high-performance computing tasks. **b** Schematic representation of sampling density for each of the seven cohort members on study, also specifying whether whole exome sequencing and/or targeted panel sequencing was performed on that sample. All samples received ultra-low-pass whole genome sequencing. **c** Tumor fraction dynamics colored by individual. Tumor fraction was measured on study using ultra-low-pass whole genome sequencing and the ichorCNA algorithm. **d** Tumor fraction dynamics recolored by RECIST v1.1 response by imaging categories. RECIST v1.1 bucket response type into several categories: complete response (CR), partial response (PR), stable disease (SD), and progressive disease (PD)

Thirty-five patients with metastatic TNBC, who were enrolled on a phase II study of cabozantinib monotherapy (NCT01738438), had available, banked, narrowly sampled, plasma-derived ctDNA samples [27]. Using ultra-low-pass whole genome sequencing (ULP-WGS) at approximately 0.1x coverage, ctDNA tumor fraction (TFx) was computational estimated using the ichorCNA algorithm [4] for each available sample. We identified seven individuals with at least three measurements of ctDNA TFx >0.10 who all had similar baseline TFx values (range 0.22–0.34). The clinical and pathologic characteristics of the selected patients mirrored those of the remaining, excluded study population (Table 1). Among the pertinent characteristics evaluated, we found no significant differences between included and excluded individuals in stage at diagnosis of primary breast cancer (*p* value: 0.74, chi-squared test), neoadjuvant therapy received (*p* value 0.24, chi-squared test), and prior lines of metastatic treatment (*p* value 0.44, chi-squared test), among others. The selected women were between 42 and 69 years old at the time of sample collection, with a median age of 52. Each patient had received neoadjuvant therapy and surgery for localized disease, then had mTNBC confirmed by metastatic biopsy. In total, there were 42 samples on seven patients (4–8 per individual, median = 6; Fig. 1a). Sample collection occurred regularly, every 6–49 days with a median time of 21 days between samples (Fig. 1b, Additional file 1: Table S1). We performed 10,000x unique molecular identifier (UMI)-based targeted panel sequencing (TPS) for each plasma sample with matched germline, and orthogonal 150x whole exome sequencing (WES) for samples with TFx >0.10, along with matched germline (Additional file 1: Table S2).

**Table 1:** Cohort clinical and pathologic characteristics

	Current clonal dynamics	Remaining patients	***P*** value
	Study cohort	In phase II study	
**Age**			0.67
Median	52	49	
Range	42–69	31–78	
**Stage at diagnosis of primary breast cancer**			0.74
I	1 (14%)	5 (17%)	
II	4 (57%)	15 (50%)	
III	1 (14%)	7 (23%)	
Iv	1 (14%)	1 (3%)	
**Germline BRCA status**			0.97
Wildtype	6 (86%)	20 (71%)	
Mutant	1 (14%)	4 (14%)	
Unkown	0 (0%)	4 (14%)	
**Primary cancer receptor status**			0.25
Triple negative	5 (72%)	23 (82%)	
**Metastatic biopsy receptor status**			1
Triple negative	7 (100%)	28 (100%)	
**Metastatic site**			
Lung metastases	1 (14%)	13 (46%)	0.1
Liver metastases	4 (57%)	7 (25%)	0.11
Bone metastases	5 (71%)	12 (43%)	0.17
**Neoadjuvant therapy**			0.24
Recieved	7 (100%)	19 (68%)	
**Prior lines of metastatic therapy**			0.44
0	1 (14%)	5 (18%)	
1	4 (57%)	14 (50%)	
2	1 (14%)	3 (11%)	
3+	1 (14%)	6 (21%)

### Circulating tumor DNA content fluctuates during treatment

Estimates of ctDNA tumor fraction computed from ULP-WGS using the ichorCNA package showed considerable variation during treatment, including crossing below the threshold of 0.10 tumor fraction (Fig. 1c), which has been shown to be associated with overall survival in mTNBC [11]. Tumor fraction ranged from 0.025 to 0.443 with a median value of 0.18. In the first 8 days of cabozantinib treatment, the phase-II cohort displayed a significant reduction in tumor fraction (paired two sample *T* test, *D* = −0.056, 95% CI [−0.089, −0.022], *p* value=0.002) (Figure S1). We evaluated the magnitude and direction of change from cycle 1, day 1 of treatment (C1D1) to cycle 1, day 8 (C1D8), and its association with best imaging response via RECIST v1.1 [38] and Choi CT criteria [39]. We modeled the relationship via logistic regression and found no significant relationship between initial tumor fraction change and RECIST/Choi measured outcomes (*p* value = 0.59 and 0.69 for RECIST and Choi criteria, respectively; Additional file 1: Figure S1 B-C). Within the seven-patient cohort, we found similar discordance between tumor fraction dynamics and imaging response: all patients had stable disease as best RECISTv1.1 imaging response despite significant variation in TFx with some patients’ TFx rising and others demonstrating significant decline (Fig. 1d).

### Orthogonal ctDNA sequencing approaches are highly concordant

**Fig. 2:**
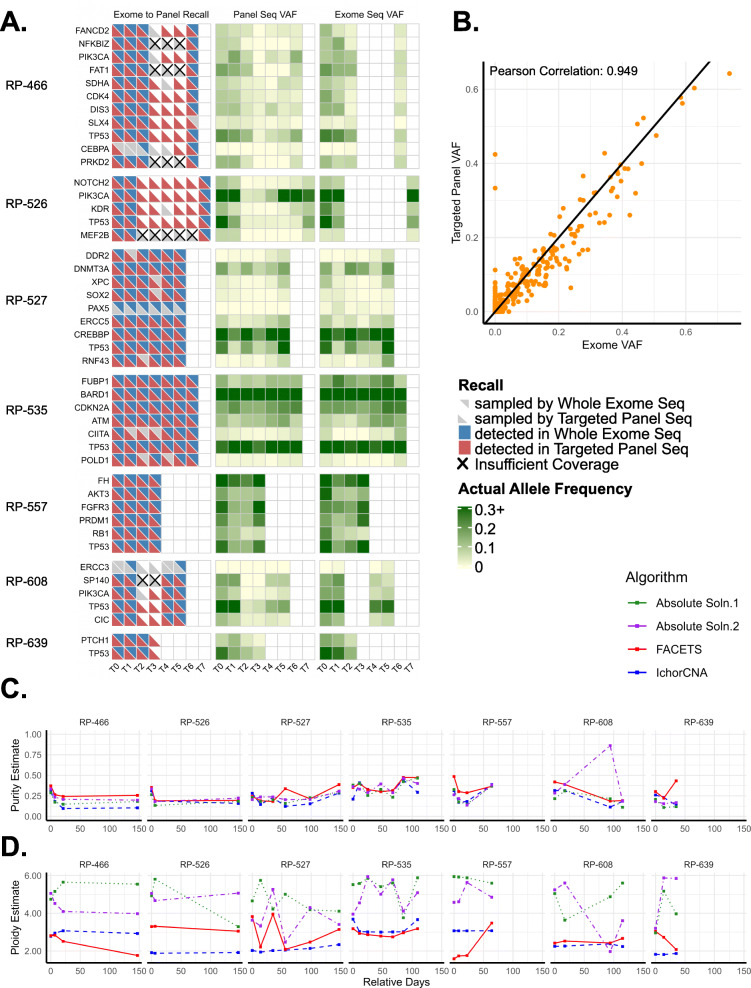
Orthogonal ctDNA sequencing approaches are highly concordant. Somatic SNV and INDEL calling of whole exome sequencing (WES; average depth 150X) and targeted panel sequencing (TPS; nominal sequencing depth 10,000X) were completed on the Terra/Firecloud platform using gatk-Mutect2 pipelines (McKenna et al., 2010). **a** Variant recall assessment of TPS on somatic variants discovered in one or more WES assays. Only variants intersecting theoretical capture regions of TPS were considered. Variants used in assessment were those called in WES at any point, which also overlapped in genomic position with target or bait regions included in the TPS. X’s indicate a lack of adequate sequencing depth in the TPS. Center and right panels compare variant allele frequency (VAF) data from each assay. **b** Scatter plot comparing estimated VAF in TPS and WES sequencing across all individuals and time points. 1:1 line drawn for reference. **c** WES and ULP-WGS based algorithmic estimates of sample purity (a.k.a. tumor fraction) across samples and time points with high tumor fraction (TFx > 10%). **d** Algorithm estimation of ploidy (averaged copy number state across genome) across WES and ULP-WGS-based methods at time points with high tumor fraction. ABSOLUTE Soln.1 and Soln.2 represent the top two proposed solutions by model likelihood (Included here, as ABSOLUTE often suggests manual curation and/or override of the top solution)

Published reports vary in the concordance of ctDNA single-nucleotide variant (SNV) detection across orthogonal sequencing approaches—from very high concordance [4] to relatively poor concordance, even across commercial platforms [40]. To address this, we assessed whether ctDNA TPS can recapitulate variants detected via WES. In our selected cohort, we identified 45 somatic alterations which were called by Mutect2 in one or more WES experiments and also intersected the genomic intervals captured in the targeted panel sequencing. Using this set of observed alterations, we searched for support in our targeted panel sequencing, in order to measure agreement in the two sequencing modalities. In general, we found that the recall of WES events in TPS was very high, sensitivity = 0.955 and reliable across time (Fig. 2a). In our variant set, TPS detected more somatic calls than WES especially at low variant allele frequencies (VAF), not unexpected due to the higher achievable sequencing depth in combination with the UMI-based read processing protocol, which reduces false positive results. For each site in the test set, we compared VAF between WES and TPS and found that VAF measurements were highly concordant (Pearson’s *r*=0.949) (Fig. 2a, b). Collectively, these data demonstrate that orthogonal TPS and WES sequencing approaches demonstrate robust concordance in both SNV detection and VAF among shared loci.

Accurate measures of purity and ploidy are crucial to modeling tumor evolution and subclonal structure. To evaluate estimation methods for purity and ploidy, we employed three popular, open-source, orthogonal methods designed for either WES or ULP-WGS data and compared across time points with high tumor fraction (TFx > 10%). We ran ABSOLUTE [31] and FACETS [32] on WES data and compared the results to estimates provided by ichorCNA [4] run on ULP-WGS data. We found that purity measurements were generally robust to differences in algorithm and sequencing modality (Fig. 2c). However, ploidy solutions were less stable (Fig. 2d), even across samples drawn over a short timeframe from the same patient, among which one would not anticipate a significant shift in ploidy. Overall, ichorCNA provided the most stable ploidy profile, with similar purity estimates to ABSOLUTE/FACETS. For subsequent modeling of clonal structure, we used ichorCNA purity and ploidy solutions.

### Copy number profiles are stable during treatment for metastatic breast cancer

**Fig. 3.**
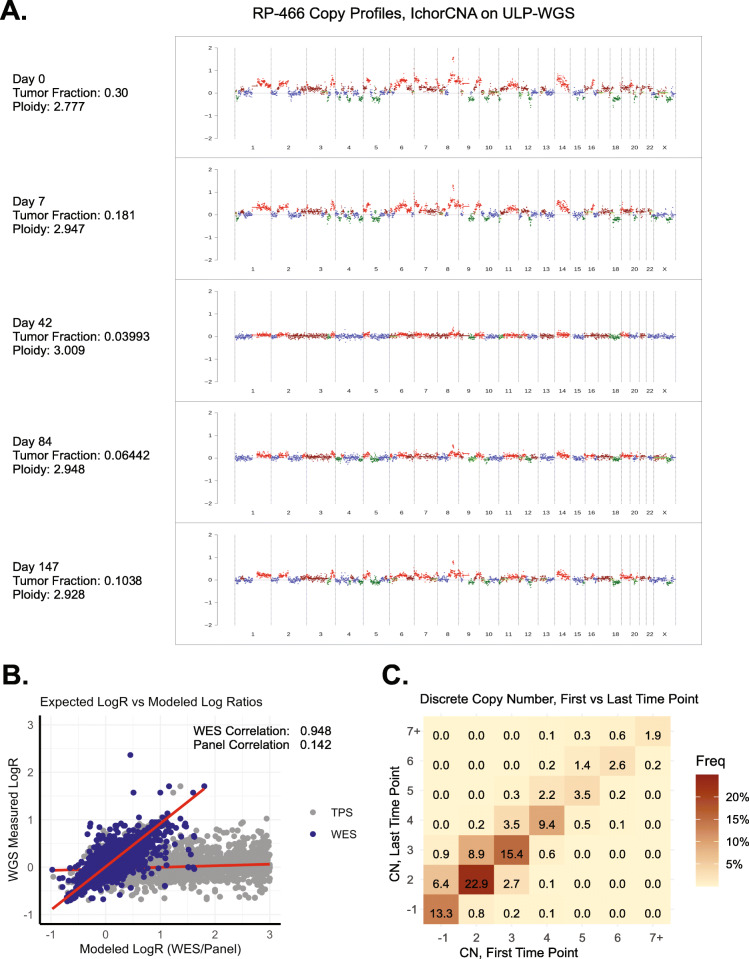
Copy number profiles are stable. Ultra-low pass whole genome sequencing (ULP-WGS) was performed on all 42 ctDNA samples and tumor fraction and copy number data derived using ichorCNA. **a** Genome-wide copy profile of patient RP-466, derived from ULP-WGS on liquid biopsy ctDNA, showing changes in focal event resolution resulting from shifts in tumor fraction. Dark green segments represent a copy number of 1; blue represent neutral or 2 copies, brown and red represent 3 and 4+, respectively. **b** Scatter plot of computed log-ratios in ULP-WGS, compared to those derived from WES or TPS data using binned read-count of on and off target bins. **c** Discrete copy number confusion matrix for ULP-WGS based calls at first and last time points. All samples had tumor fraction ≥10%. Genomic positions assayed between first and last time points were uniformly and randomly sampled, and discrete copy number states were capped between one and seven during initial ichorCNA analyses

ULP-WGS of ctDNA provides high-quality copy number information at TFx>0.10, making it feasible to follow somatic copy number alterations (SCNAs) over the course of treatment in the metastatic setting. Using ichorCNA copy number profiles, we examined longitudinal changes in log ratio and copy number state. Reductions in TFx corresponded with lower resolution copy number profiles, as evident in the case vignette of participant RP-466 (Fig. 3a). At the lowest levels of TFx, global trends in somatic copy number alterations (SCNAs) were maintained, but focal and sub-arm level chromosomal events, like those at 1p, 4q, 10p, and 12q loci, were lost. These trends were largely mirrored in the profiles of the other cohort patients (Additional file 1: Figure S2).

To understand the SCNA dynamics, we assessed SCNA stability between the first and last sequencing time points for each patient in the cohort. We randomly and uniformly sampled genomic positions, querying their states at the first and last time points, and constructed a confusion matrix of possible copy number states (Fig. 3b, c). Overall, we found stable genome structure from first to last sampled time point, with SCNA calls collapsed into amplification, neutral, and deletion states (balanced accuracy = 0.858, sensitivity = 0.815, specificity = 0.900). Similarly, comparison of the discrete copy number at the first and last time point per patient also yielded high accuracy, sensitivity, and specificity (balanced accuracy = 0.830, sensitivity = 0.716, specificity = 0.945), implying stability of the more specific, called states over time.

To test the coherence of copy number information provided by ctDNA WES and TPS, we compared the log ratios computed from ULP-WGS and the corresponding measurement from either WES or TPS (target reads only). WES displayed high concordance to ULP-WGS estimates of log ratio (Pearson’s *r* = 0.948), but TPS displayed very little relationship to ULP-WGS (Person’s *r* = 0.148) (Fig. 3b, c). In terms of the collapsed copy states (i.e., amplification, neutral, and deletion), WES predicted ULP-WGS states at rates better than chance (balanced accuracy = 0.746, sensitivity = 0.663, specificity = 0.830). TPS predicted these same states no better than random chance (balanced accuracy = 0.523, sensitivity = 0.364, specificity = 0.682). It may be the case that the on-target/off-target binned read count strategy used in the WES/TPS copy number analyses may be improved through the incorporation of allelic imbalance information at common SNP loci if targeted panel bait sets are appropriately designed.

### Modeling clonal architecture over narrow time frames via ctDNA

As ctDNA offers the potential for high density, minimally invasive sample collection, we explored its ability to model the clonal structure of metastatic disease progression. Combining somatic variants found by deep TPS, as well as total copy number information, purity, and ploidy from ULP-WGS, we modeled the tumor subclonal structure using the PyClone software package [34]. PyClone uses a hierarchical Bayesian approach, allowing for joint estimation across variants and time points [34]. PyClone assigns variants into clusters, representing underlying cellular populations or clones, and estimates corresponding adjusted cellular prevalence for each clone within the tumor proportion. Using a combinatorial approach which interfaces easily with PyClone output profiles [35], we built phylogenetic trees and labeled the detected non-synonymous, somatic alterations.

**Fig. 4:**
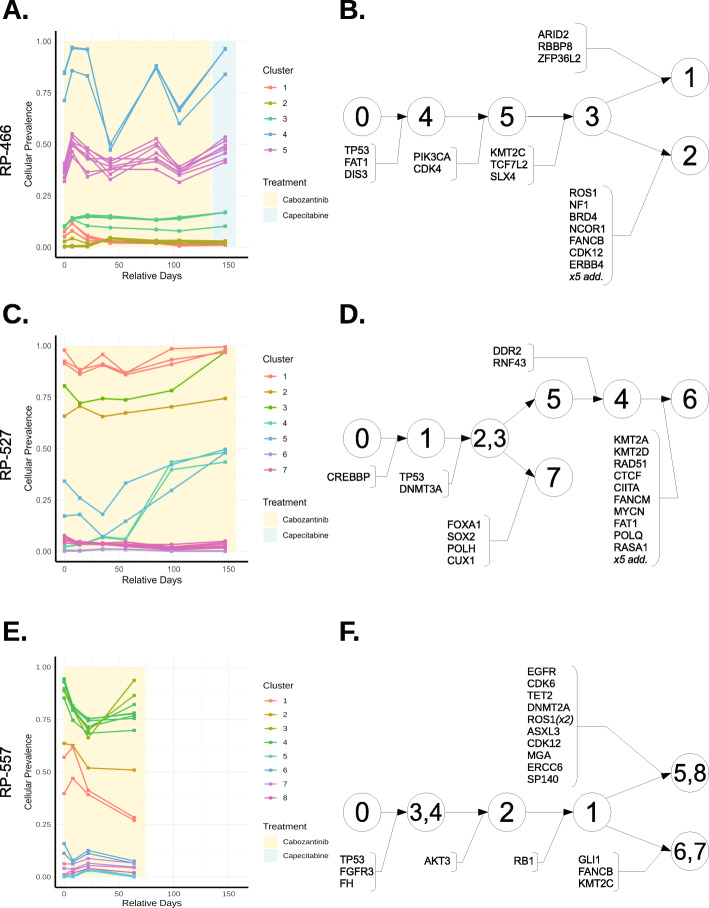
Tumor subclonal dynamics vary across patients. Models of clonal and subclonal populations which make up the cancers of metastatic patients, derived using PyClone [34]. Variant inputs include union of filter-passing alterations from each sampled time point delivered by the commercially available liquid-biopsy targeted panel-sequencing pipeline at the Broad Institute. Copy number information and purity were derived from ichorCNA. **a**, **b** Clonal prevalence dynamics, clustering, and inferred phylogenetic tree structure for patient RP-466, revealing generally unchanging populations in the tumor, with important drivers occupying early positions in cell lineages. **c**, **d** RP-527 clonal dynamics profile and inferred tree structure showing statistically significant clonal expansion of cell lineage marked by non-synonymous DDR2 and RNF43 variants. **e**, **f** RP-557 profile and tree showing the opposite trend as RP-527, with a decreasing cell population marked by RB1 mutation

In our cohort, the structure and dynamics of subclonal populations varied considerably. Profiles of three patients, RP-466, RP-527, and RP-557, illustrate the observed trends among the patients (Fig. 4). RP-466 clone populations were characterized, generally by stability across the 147-day sampling window (Fig. 4a).

We noted that RP-466’s profile appears to fluctuate at the fourth and sixth time points, in discordance with the rest of the profile, potentially revealing overestimation of sample purity at those time points. As an added sensitivity analysis (Additional file 1: Figure S3), we re-ran the PyClone model under the same conditions removing (1) the sample corresponding to the fourth time point (Additional file 1: Figure S3A) and (2) the samples corresponding to the fourth, fifth, and sixth time points (Additional file 1: Figure S3B). The removal of these samples has little impact on the general trends of the clonal structure, with both plots display the same hallmark stability, as the original analysis. We note the only identifiable change occurs in the second of the two sensitivity analyses, where low-level residual clusters inconsistently split into an additional group. Since our principal intent is to assess the feasibility of this modeling approach to real-time monitoring, we feet the removal of any of these samples may tend to misrepresent the anticipated utility of this approach; thus, we decided to keep the original data intact for subsequent analyses (Fig. 4a). The phylogenetic tree structure inferred for patient RP-466 features hallmark characteristics of cancer evolution, with high prevalence somatic alterations in tumor suppressors and pleiotropic signaling pathway members; *TP53*, *CREBBP*, *PIK3CA*, and *CDK4*. Subsequently appearing non-synonymous alterations span a wide variety of biological processes. It’s unclear whether any of these variants assigned to lower nodes play important roles in subclone identity or whether they are passenger mutations.

In contrast, RP-527 and RP-557 reflect shifts in clonal dynamics over narrow time windows. In RP-527, cluster four significantly expanded from background prevalence levels (D147-D0 = 0.461, *n* = 2, *p* value = 0.04552, Welch two-sample *T* test) and persists for at least 49 days (Fig. 4c). This expanding cluster contained two missense variants of consequence, a K/N substitution in the receptor tyrosine kinase *DDR2* as well as a splice-site variant in the tumor suppressor *RNF43* (Fig. 4d). DDR2 is a known target of cabozantinib, shown to be inhibited through kinome analysis of cabozantinib clinical trial specimens via quantitative kinome analysis [41]. On the other hand, RP-557 demonstrates a subclone (cluster 1) that drops in prevalence (D64-D0 = −0.205, *n* = 2, *p* value = 0.1732, Welch two-sample *T* test), over the 64-day period. This drop appears to co-occur with the dynamics in the dominant clones represented by clusters 3 and 4 which dip and then rise in the final sampled time point (Fig. 4e). This cluster is characterized by decreased prevalence of a missense mutation encoding a single H/L substitution in exon 11 of the tumor suppressor *RB1* (Fig. 4f). Clonal dynamics for the remaining patients are visualized in (Additional file 1: Figure S4) as are all variants and clonal abundances for all samples (Additional file 1: Figure S5), with variants annotated in (Additional file 2: Table S3), both of which were completed using the same TPS/ULP-WGS modeling strategy.

### Whole exome sequencing uncovers driver mutations and allows neoantigen discovery

**Fig. 5:**
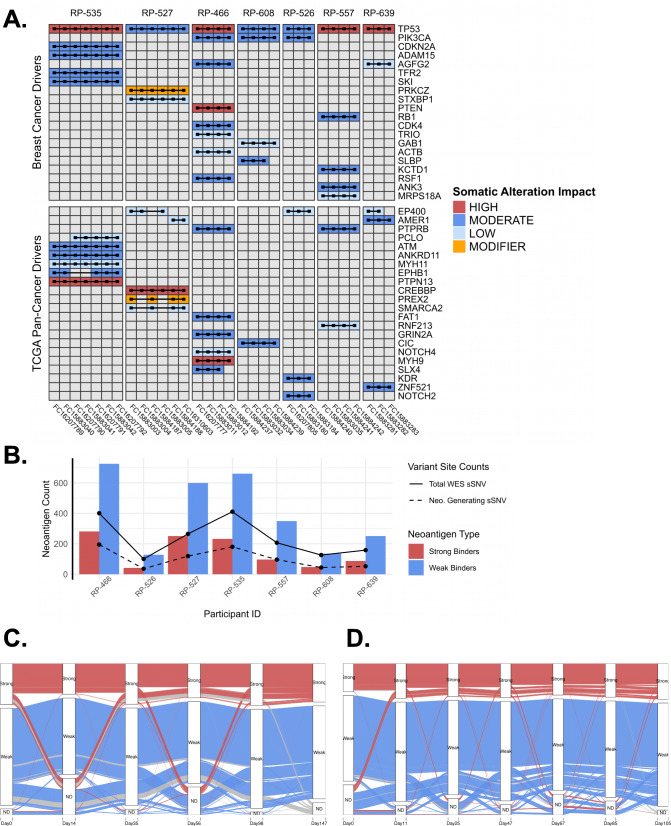
Whole exome sequencing uncovers driver mutations and allows neoantigen prediction. Whole exome sequencing results from 31 total samples with tumor fraction ≥10% using short variant and INDEL calling tools from gatk-Mutect2 pipelines (McKenna et al., 2010), with subsequent neoantigen binding predictions for known MHC molecules from NetMHCpan 4.0 (Reynisson et al., 2020). **a** Driver mutations found via whole exome sequencing across time points. Variant data visualized are those whose genes have been previously annotated in literature as breast cancer drivers or pan cancer drivers. **b** Trends in predicted neoantigens among cohort members. Strong binders are denoted as those peptide sequences with NetMHCpan ranks <0.5%, and weak binders are those with ranks <2%. Neoantigen Generating sSNV are alterations whose changes to peptide structure are predicted to produce neoantigens capable of strong or weak binding to known MHC molecules. **c**, **d** Neoantigen dynamics from patient RP-527 and RP-535, showing proportions of detected neoantigens and dropout over time. Strong, weak, and ND labels correspond to binding affinity of predicted neoantigens, as well as a non-detected category to capture dropout. Threads are colored by their state at the final sequencing time point

To examine the longitudinal consistency of driver gene variant calling in ctDNA WES data, we looked at known driver mutations previously outlined in the breast cancer literature [42, 43] as well as those found by the TCGA Pan Cancer Atlas studies and OncoKB [44, 45]. Our data indicate that WES of ctDNA samples recovers driver variants consistently over multiple time points (Fig. 5a). For example, the most frequently altered genes were TP53 and PIK3CA, detected at every time point in seven and three cohort members, respectively. Among pan cancer drivers, EP400 was detected in three individuals, and both synonymous and non-synonymous alterations in the genes AMER1 and PTPRB were detected in two cohort members. The low dropout rates of variants over up to seven consecutive exomes at moderate read depth indicate that detection of driver mutations overtime with ctDNA is feasible.

Whole exome sequencing of ctDNA allows computational prediction of neoantigens. To this end, we leveraged NetMHCpan 4.0 [36], a published tool for neoantigen prediction from mutational data. Among the patients in our cohort, we detected between 36 and 195 novel alterations (median = 96) predicted to produce either strongly or weakly binding neoantigens (Fig. 5b). These sites account for 182–1007 unique peptide presentations per individual (median = 445). In general, we found the number of novel, neoantigen-producing alterations have a strong and positive correlation with the total mutation burden (Pearson’s *R* = 0.992). Weakly binding neoantigens were predicted more often than strongly binding neoantigens, with an average detection ratio of 2.9:1.

To take advantage of the serial nature of our study, we looked at neoantigen dynamics over time. Representative trends are illustrated by the individual profiles of RP-527 (Fig. 5c) and RP-535 (Fig. 5d). Predicted neoantigen dynamics for the other patients are visualized (Additional file 1: Figure S6). Notably, the majority of strongly and weakly binding neoantigen producing alleles are detectable at all time points (RP-527 = 475/851, RP-535 = 530/893 omnipresent neoantigens), despite fluctuations in tumor fraction and clinical response. Despite this trend, not all neoantigens are present at baseline. In RP-527 and RP-535, we found 4.1% and 20.8% of variants resulting in neoantigens were totally absent in day zero sequencing. In RP-527, we find neoantigens which appear at the initial and final time points but disappear intermittently below detectable levels in mid-series sequencing events. In both profiles, we also find neoantigen alleles which dropout without re-detection, possibly indicating loss of specific cell populations harboring these variants. In addition, we find potentially clinically important patterns of dynamics in specific neoantigens which only present later in the course of therapy. In RP-535, for instance, we find specific neoantigens which present in the sixth and seventh exome sequencing assays, suggesting the development or expansion of variants resulting in novel predicted neoantigens.

In an exploratory analysis, we investigated the association of the number of neoantigens with clinical outcomes in this small cohort. We found no significant association between cabozantinib progression-free survival (PFS) and total neoantigens either categorically (above vs. below median total neoantigens, log-rank *p*=0.87; Additional file 1: Figure S7A) or continuously (per 10 total neoantigens: HR 1.002, 95% CI 0.98–1.02, log-rank *p*=0.9). However, there was an association between overall survival from metastatic diagnosis and total neoantigens, both categorically (above vs. below median total neoantigens, log-rank *p*=0.02; Additional file 1: Figure S7B) and continuously (per 10 neoantigens: HR 0.96, 95% CI 0.92–1.0, log-rank *p*=0.03). This relationship between overall survival and neoantigens remained significant for both strong binders alone (per 10 neoantigens: HR 0.86, 95% CI 0.72–1.03, log-rank *p*=0.03), and weak binders alone (per 10 neoantigens: HR 0.95, 95% CI 0.89–1.01, log-rank *p*=0.04). These exploratory data support investigation of predicted neoantigens in larger cohorts of mTNBC with multivariable models.

## Discussion

While tumor biopsies remain the gold standard for diagnosis, ctDNA-based “liquid biopsies” overcome many limitations of tumor biopsies: metastases may be inaccessible or not feasible to biopsy serially over time [46, 47]; biopsies sample a localized region of a single metastatic site, which may introduce sampling bias [48]; biopsies may be painful and cause anxiety; and biopsies have a risk of bleeding or infection [46]. Minimally invasive ctDNA assays from simple blood draws offer the potential to serially analyze tumor genomic features through a more patient-centric approach. To date, our understanding of the opportunities and limitations of frequent ctDNA analyses over days to weeks via orthogonal sequencing approaches is limited. In this study, we sought to understand (1) tumor genomic changes (SNVs, SCNAs, predicted neoantigens) detectable over narrow time windows and (2) the performance, utility, and limitations of orthogonal sequencing approaches and algorithms on serial ctDNA samples.

This study provides an important assessment of the tumor genomic features that change, or remain stable, over narrow time windows. Overall, copy number was stable across the seven patients in this cohort. This may reflect that large-scale SCNA events occur early in TNBC development and subsequent alterations are infrequent [49, 50], but should be evaluated in other tumor types and settings (e.g., DNA damaging chemotherapy). Alternatively, this may reflect challenges and limitations of SCNA characterization via ctDNA (discussed further below).

Conversely, we detected shifts in SNVs both via TPS and WES approaches. To track within-patient clonal dynamics, we evaluated a combined ULP-WGS + TPS approach to obtain purity, ploidy, copy number, and variant data, using PyClone for clonal reconstruction. In general, performance of PyClone and subsequent phylogenetic reconstruction appear to depend heavily on the number of variants recovered, and the number of samples taken. Our profiles with the best resolution had a high number of variants and many time points. Deeper sequencing may increase the number of trackable variants recovered from ctDNA, as well as lower the error in modeling prevalence. Additionally, joint modeling across sampling events is beneficial for studying response or resistance to treatments over time. Finally, there were many low prevalence variants (VAF< 20%), which were inconsistently recovered across time. Further resolution of these low-prevalence variants may be possible with deeper sequencing, or deep sequencing of paired WBC, depending on whether these markers represent members of the tumor cell phylogeny or contaminating artifacts of clonal hematopoiesis. Recent advances in personalized ctDNA-based assays, in which a patients’ tumor is sequenced and validated mutation-specific primers are developed, may allow for higher sensitivity detection of known variants in ctDNA than the method used in this study [20, 22, 51]. However, these personalized mutation panels fail to capture the development of new alterations over time, limiting their utility to largely retrospective analyses.

An intriguing finding regarding predicted neoantigens from this study was the detection of either newly developed or clonally expanding alterations that result in predicted neoantigens over relatively short time frames. This is the first study, to our knowledge, to specifically track shifts in predicted neoantigens via ctDNA within individual patients. Knowledge of sustained and emerging neoantigen peptides has potential implications for immunotherapy, including neoantigen vaccine development and selecting patients or optimizing tumors to respond to checkpoint inhibitors.

This study is unique in analyzing serial ctDNA samples via multiple ctDNA sequencing modalities (ULP-WGS, TPS, WES). A major hurdle to clinical implementation of ctDNA sequencing is inconsistency across platforms [40, 52]. When evaluating specific alterations that demonstrated adequate sequencing quality and coverage on both TPS and WES, we found very high recall. This reinforces the importance of clinical ctDNA sequencing assays reporting quality metrics. While reliable total copy number information can be inferred in most ctDNA samples via ULP-WGS, allele-specific SCNA resolution, especially for exome-based or panel-based assays, remains a challenge. Other future areas of investigation include determinants of ctDNA “shedders” versus “non-shedders” and the best use of very low TFx samples. Additionally, we believe that investigating approaches specific to copy number analysis on liquid biopsy exome and panel sequencing would allow for more precise and affordable genetic monitoring in metastatic cancers. Further work will also explore pre-analytical and analytical factors impacting ctDNA results, particularly for rare variants detected at low allele fractions.

This study does have limitations. Given the multi-sample, orthogonal sequencing analysis approach, we focused on a small number of patients with a single cancer sub-type who all received the same therapy on clinical trial without a clinical response. Thus, while we make some fundamental observations on technical aspects, generalization will require larger studies in other tumor types and clinically interesting settings. Also, these samples were collected using EDTA tubes rather than the common preservative-based tubes commonly used for ctDNA studies now: however, processing conformed to ASCO/CAP guidelines [53].

## Conclusions

In this work, we demonstrate that analysis of multiple ctDNA samples collected from patients over narrow windows of time is not only feasible, but provides potentially important insights into clonal and neoantigen dynamics. Our approach reveals strengths and limitations of existing ctDNA sequencing and analytical approaches. In the future, we anticipate the expansion of ctDNA applications in clinical use, including serial genetic monitoring of tumor dynamics in metastatic patients, neoantigen prediction for immunogenic therapies, and real-time modeling of prognoses. Our hope is that low cost, minimally invasive genetic monitoring, made possible through ctDNA profiling, expands the toolkit of physicians and patients in metastatic cancers of all types—allowing more responsive approaches to the management of metastatic treatment and facilitating novel methodologies in translational research.

## Supplementary Information

**Additional file 1.** Supplementary Figures and Tables, excluding Table S3.

**Additional file 2.** Supplementary Table S3.

## Data Availability

All sequencing data supporting the conclusions of this paper are deposited to dbGAP, dbGaP Accession Number: phs001417.v2.p1. While awaiting data release via dbGaP, investigators may contact the corresponding author to discuss gaining access to the data. Data and code from downstream analyses are available through a Gitlab repository (https://gitlab.com/Zt_Weber/narrow-interval-clonal-structure-mbc.git) [54].

## Abbreviations

cfDNA: Cell free DNA. DNA of any origin, found suspended in the liquid component of blood
ctDNA: Circulating tumor DNA. cfDNA of tumor origin
PCR: Polymerase Chain Reaction. Experimental technique used to amplify isolated DNA sequences
RECIST: Response Evaluation Criteria in Solid Tumors. Guidelines for Imaging based assessment of treatment response in solid cancers
TFx: Tumor Fraction (a.k.a. sample purity). The fraction of cfDNA in a sample originating from the tumor
TNBC: Triple Negative Breast Cancer. Aggressive subtype of breast cancer characterized by lack of HER2, ER, and PR molecular targets
TPS: Targeted Panel Sequencing. Sequencing assay which assesses a set of gene targets with known importance to the disease of interest (here, cancers in general)
(S)CNA: (Somatic) Copy Number Alterations. Regions of the genome which have undergone structural changes, I.e. duplication or deletion, sometimes repeatedly
(s)SNV: (Somatic) Single/Small Nucleotide Variants. Mutations in DNA sequences which arise as the result of DNA damage and subsequent repair. Traditionally, SNV events typically involve a single nucleotide, or a small number of nucleotides
ULP-WGS: Ultra Low Pass Whole Genome Sequencing. Genome sequencing assay targeting approximately 0.1x per-base coverage, useful in global copy number and purity/ploidy estimation
WBC: White Blood Cells. Nucleated cells of hematopoietic lineage. Components of the immune system
WES: Whole Exome Sequencing. Sequencing assay which aims to generate reads covering all of the coding sequences in the genome, useful in detecting mutations which change the structure and function of native proteins
