# Supplementary material for: Modeling clonal structure over narrow time frames via circulating tumor DNA in metastatic breast cancer
Source: Genome Med. 2021 May 20;13:89. doi: 10.1186/s13073-021-00895-x (PMC8136103; doi:10.1186/s13073-021-00895-x)
Supplement: Supplementary file 1 — Additional file 1. Supplementary Figures and Tables, excluding Table S3. [file 13073_2021_895_MOESM1_ESM.docx]

**Supplementary tables and figures**

**Modeling clonal structure over narrow time frames via circulating tumor DNA in metastatic breast cancer**

**Table S1: Sample Capture Information for 7 Patient cohort.** ctDNA was isolated and later sequenced from serum plasma banked during the original Phase-II Trial (S.M. Tolaney, et al., 2017) of Cabozantinib in Metastatic TNBC. Table contains individual sample IDS, a list of assays performed on each sample, relative days from treatment initiation, estimated tumor fraction of each sample, and current therapy at time of treatment. **ULP**: Ultra-low pass whole genome sequencing; **WES**: Whole exome sequencing; **PANEL**: Targeted Panel Sequencing; **TFx**: Tumor fraction; **Cabo**: Cabozantinib monotherapy; **Cap**: Capecitabine monotherapy.

**Table S2: Sequencing Metrics by Assay Type**

Average read depth achieved for each sequencing assay type. For whole exome sequencing (WES), rage of achieved depths provided. For ultra-low pass whole genome sequencing (ULP-WGS), range of coverage reported. For targeted panel sequencing (TPS), nominal read depth was approximately 10,000X; final read depth after deduplex reported.

**
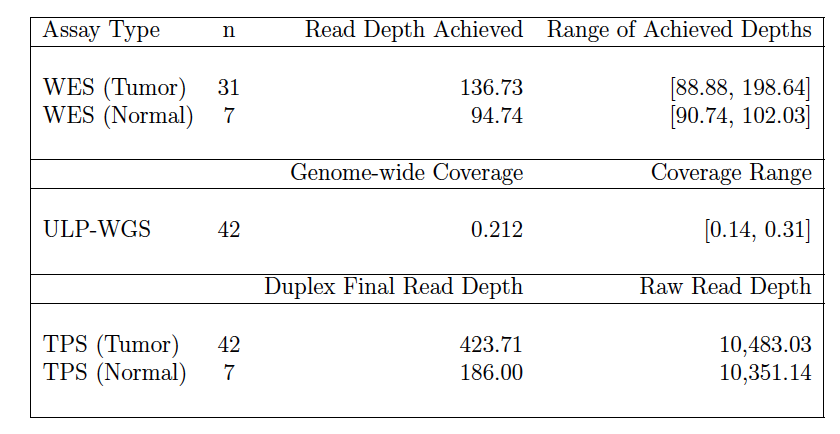
**

**Fig. S1: Change in Tumor Fraction from Day 1 to Day 8.** Tumor fraction was calculated for each patient on the cabozantinib study (n=35) on cycle 1-day 1 (C1D1) and cycle 1-day 8 (C1D8; +/- 1 day). There was a significant decline across all samples (**A**). The association between C1D1:C1D8 change and response to imaging defined by RECISTv1.1 (**B**) and Choi (**C**) methodologies are visualized.

**Fig S2: Copy Number Plots for All Timepoints.** Copy number log ratio plot from optimal solution via ichorCNA presented for each timepoint for each patient. Bright red indicates amplification, dark red low gain, blue copy neutral, and green loss. Tumor fraction (TFx) and ploidy noted.

**Fig S3: Sensitivity Analyses of Clonal Abundance for Patient RP-466.** As an added sensitivity analysis to evaluate clonal abundance estimates for patient RP-466 due to point-to-point variability, we re-ran the PyClone model under the same conditions removing (A) the sample corresponding to the fourth time point, and (B) the samples corresponding to the fourth, fifth, and sixth time points.

1. PyClone plot with only time point 4 removed
2. PyClone plot with time points 4/5/6 removed

**Fig S4: Tumor sub-clonal dynamics vary across patients across similar time spans and treatments.** To model the clonal structure and dynamics of the cancers of metastatic patients, we employed the popular python-based tool, PyClone, to use hierarchical-Bayes techniques for jointly estimating prevalence of somatic alterations and simultaneously clustering them into groups representing the underlying cancer’s cell population structure. Variant sets included union of filter-passing alterations from each sampled time point delivered by the commercially available liquid-biopsy targeted panel sequencing pipeline at the Broad Institute. Copy number information and ploidy were derived from ichorCNA. Clonal prevalence dynamics, clustering, and inferred phylogenetic tree structure for patients RP-426 (**A)**, RP-535 (**B)**, RP-608 (**C)**, and RP-639 (**D).**

**Fig S5: Variants Detected by Targeted Panel Sequencing**. PyClone was used to jointly estimate prevalence of somatic alterations and simultaneously clustering them into groups representing the underlying cancer’s cell population structure. Variant sets included union of filter-passing alterations from each sampled time point delivered by the commercially available liquid-biopsy targeted panel sequencing pipeline at the Broad Institute. Copy number information and ploidy were derived from ichorCNA. Variant cellular prevalence (Cell Prev.) are indicated by shading. Reported clusters are PyClone-defined and mirror PyClone line plots (Figure 3 and Supplemental Figure 2). Each column represents the relative days, indicated at bottom of plot.


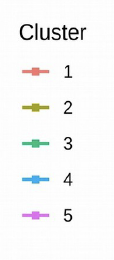

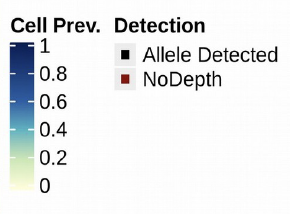


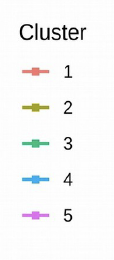


**Fig S5 (cont):**


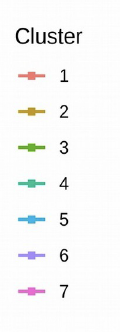

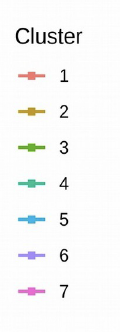


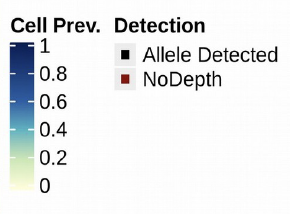


**Fig. S5 (cont):**


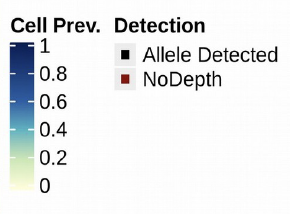

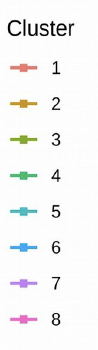


**Fig. S5 (cont):**


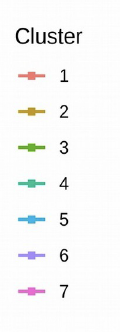

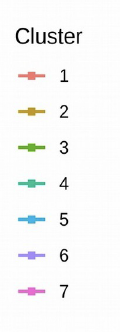


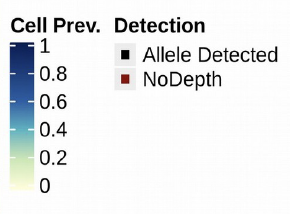


**Fig. S6: Predicted Neoantigens via ctDNA Over time.**

Whole exome sequencing was performed on 31 total samples that had tumor fraction >=10%. Short variant and INDEL calling in both WES and TPS were completed on the Terra/Firecloud platform using gatk-Mutect2 pipelines. Neoantigen binding predictions for known MHC molecules were completed using machine learning approaches learned on peptide-affinity data, NetMHCpan 4.0. Global trends in predicted neoantigens among cohort members are visualized. Strong binders are denoted as those peptide sequences with NetMHCpan ranks < 0.5%, and weak binders are those with ranks < 2%.

**Fig. S7:** **Association of Total Predicted Neoantigens and Clinical Outcome**. In an exploratory analysis, we investigated the association of neoantigens with outcome within this small cohort. Total neoantigens (detected across all time points) were categorized as above or below/equal to the median (n=445 total neoantigens). We evaluated the association of above/below median with progression-free survival (PFS) on the cabozantinib clinical trial (**A)** or overall survival from metastatic diagnosis (‘metastatic survival’; **B**). P-value indicates log-rank test.
